# Gut microbiota and uveitis: exploring novel mechanisms of inflammatory ocular diseases via the gut-eye axis

**DOI:** 10.3389/fmicb.2026.1749111

**Published:** 2026-04-22

**Authors:** Qing Gao, Meng Xiong, Shunhua Zhou, Jing Lu, Baoping Ren, Qinghua Peng, Meiyan Zeng, Houpan Song

**Affiliations:** 1 Hunan Provincial Key Laboratory of Traditional Chinese Medicine Diagnostics, Hunan University of Chinese Medicine, Changsha, Hunan, China; 2 School of Traditional Chinese Medicine, Hunan University of Chinese Medicine, Changsha, Hunan, China; 3 School of Medicine, Hunan University of Chinese Medicine, Changsha, Hunan, China

**Keywords:** gut microbiota, gut-eye axis, pathogenesis, treatment, uveitis

## Abstract

Uveitis is an inflammatory ocular condition that primarily affects young adults and is often associated with systemic and autoimmune disorders. This disease primarily affects intraocular structures such as the iris, ciliary body, and choroid. Clinically, it manifests through a series of symptoms, including eye redness, pain, and blurred vision, which significantly impact the quality of life for patients worldwide. Recently, the role of gut microbiota (GM) in the immune regulation and pathogenesis of inflammatory diseases has garnered significant scientific interest. This study aimed to investigate the potential association between GM and uveitis, with the objective of demonstrating novel mechanisms underlying inflammatory ocular diseases through the emerging concept of the “gut-eye axis.” Current research suggests that gut dysbiosis may influence the immune status of distal organs via various pathways, including molecular mimicry, modulation by microbial metabolites, disruption of intestinal immune homeostasis, and compromise of the intestinal mucosal barrier. Building on these mechanisms, we further explored innovative therapeutic strategies targeting GM and its metabolites, including probiotics, prebiotics, antibiotics, immunomodulators, phage therapy, fecal microbiota transplantation, and dietary interventions. This review systematically examined the association between GM dysbiosis and uveitis pathogenesis, offering new insights and directions for future research in this emerging field and establishing a theoretical foundation for developing targeted therapies based on the modulation of the microbiome.

## 1 Introduction

**FIGURE 1:**
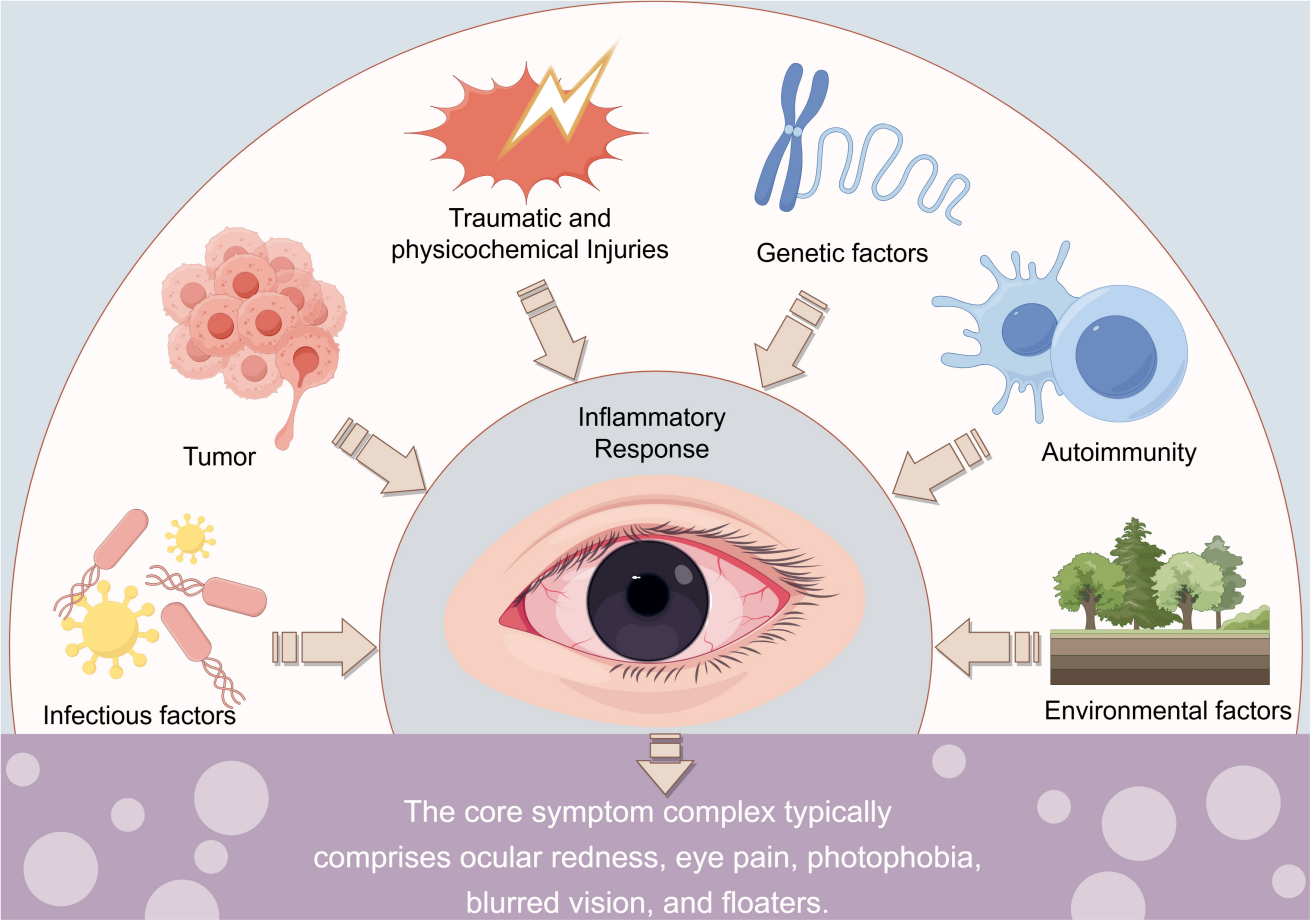
Etiology and key clinical manifestations of uveitis.

Uveitis is a heterogeneous group of potentially blinding ocular conditions characterized by intraocular inflammation (Munhoz et al., 2025). Its hallmark clinical manifestations include ocular redness, pain, light sensitivity, photophobia, floaters, and decreased vision (Liu X. et al., 2025). The etiology of uveitis is multifactorial and complex. Infections caused by pathogens such as bacteria and viruses, autoimmune diseases, primary ocular conditions, trauma or surgery, genetic predisposition, environmental influences, and malignancies all contribute to this complexity (Figure 1; Koronis et al., 2019; Mohammadi et al., 2019). Predominantly affecting young and middle-aged adults, uveitis often follows a recurrent clinical course and may lead to severe complications, such as macular edema, glaucoma, and cataracts, thereby posing a significant burden on global public health systems (Cann et al., 2018; Bai et al., 2021). Current clinical management strategies primarily involve the use of corticosteroids and immunosuppressants (Li Y. et al., 2022). However, these therapeutic agents often lack specificity in their mechanisms of action and are associated with notable systemic adverse effects when used over extended periods (Lage et al., 2023). Furthermore, some patients exhibit inadequate therapeutic responses or experience disease recurrence. Consequently, demonstrating novel molecular mechanisms underlying the pathogenesis and progression of uveitis and identifying safer and more effective therapeutic targets are critical scientific priorities that warrant urgent investigation.

Recent advances in microbiome research have revealed that the gut microbiota (GM) plays a central role in maintaining immune homeostasis as a “critical” virtual organ (Sun et al., 2022). Studies have suggested that microbial dysbiosis may be associated with the pathogenesis of multiple ocular disorders, including glaucoma (Wang et al., 2025), dry eye disease (Antman et al., 2024), and age-related macular degeneration (AMD) (Yu and Yuan, 2025), and may also be implicated in the development of uveitis. Against this backdrop, the concept of the “gut-eye axis” has emerged, offering a novel perspective for understanding inflammatory diseases that affect distant organs (Lerner et al., 2020). Accumulating evidence, largely from preclinical and animal studies, indicates that intestinal microbial dysbiosis may act as a potential mediator in the pathological processes underlying uveitis, supporting the notion that therapeutic strategies targeting the gut microbiota could represent a promising approach for the prevention and management of uveitis (Kodati and Sen, 2019).

This study provides an in-depth investigation of the interactions between GM and uveitis, with a specific focus on the characteristics of microbial dysbiosis in patients, the associations between microbial composition and different uveitis subtypes, potential mechanisms underlying the “gut-eye axis,” and emerging therapeutic strategies based on microbiota modulation. By systematically integrating the central role of the GM in uveitis pathogenesis, we highlighted the crucial importance of the intestinal ecosystem in disease development and progression. These findings are expected to significantly contribute to the development of novel therapies targeting uveitis-specific microbial profiles and related molecular biomarkers, thereby advancing the implementation of precision medicine approaches for uveitis management.

## 2 Methodology

**FIGURE 2:**
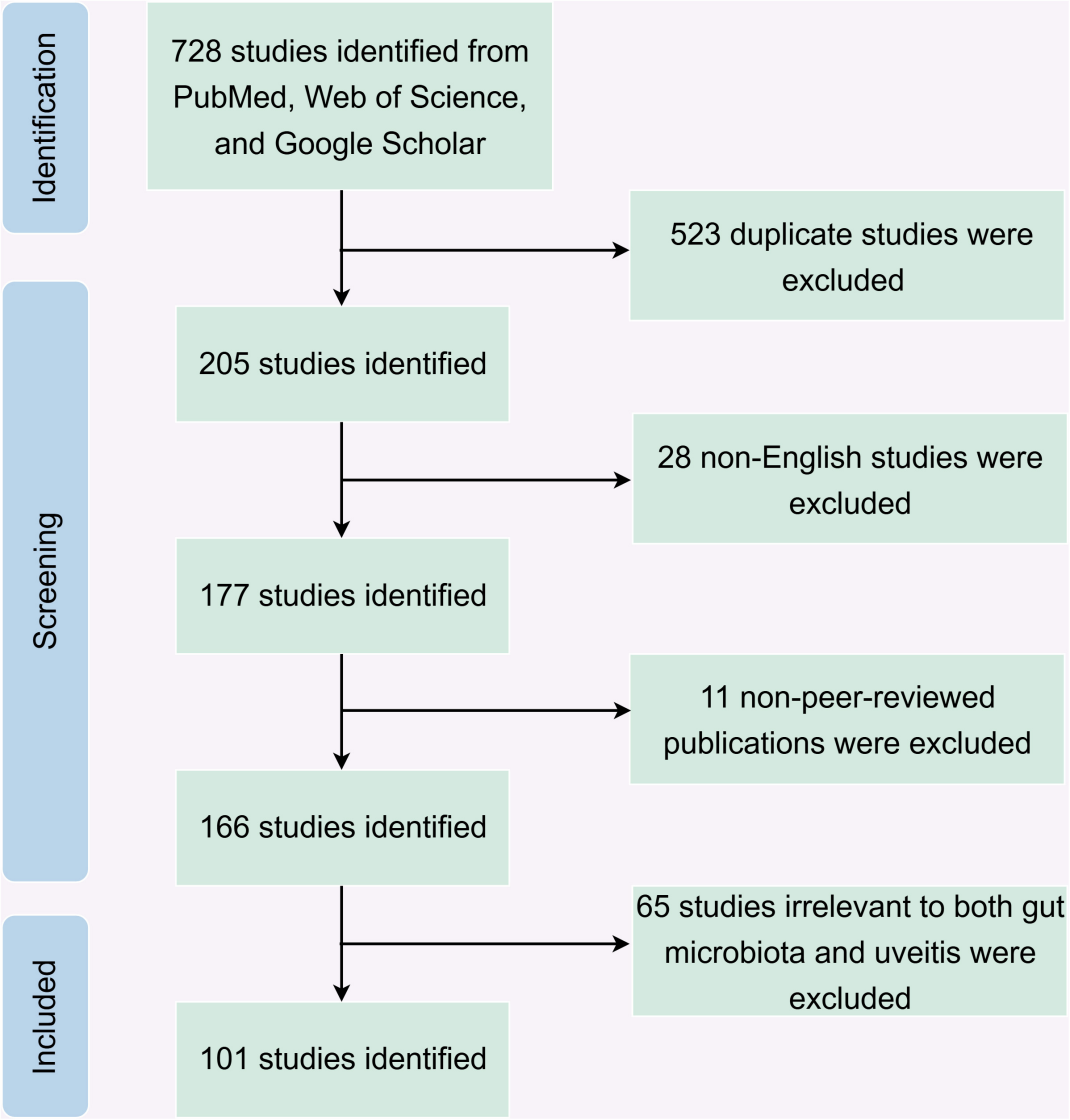
Flowchart of literature screening.

To systematically explore the relationship between gut microbiota and uveitis through the gut-eye axis, this study conducted a comprehensive literature review using databases including PubMed, Web of Science, and Google Scholar. The search strategy employed ten specific keywords: “Gut microbiota,” “Uveitis,” “Gut-eye axis,” “Autoimmune uveitis,” “Behçet’s disease,” “Vogt-Koyanagi-Harada disease,” “HLA-B27-associated uveitis,” “systemic sarcoidosis,” “ankylosing spondylitis,” “multiple sclerosis,” and “inflammatory bowel disease.” Two independent reviewers assessed each article based on predefined inclusion and exclusion criteria. The literature search covered the period from January 2010 to November 2025. The inclusion criteria were as follows: (1) articles written in English; (2) articles published in peer-reviewed journals; and (3) articles examining the association between gut microbiota, their metabolites, and uveitis-related diseases. Exclusion criteria included: (1) non-English articles; (2) non-peer-reviewed publications, such as conference abstracts, editorials, and non-academic reports; (3) articles not related to gut microbiota or uveitis; and (4) duplicate articles. The literature screening flow diagram is shown in Figure 2.

## 3 Role of gut microbiome in human health

The human gut microbiome is a diverse and dynamic microbial ecosystem that is home to approximately a trillion microorganisms and constitutes the largest microbial community in the body. Its total genomic content far exceeds that of the human genome and is considered the “second genome” of humans (Brigatti and Azaele, 2025; Dey, 2025). This system comprises various microbial taxa, including bacteria, fungi, viruses, protozoa, and archaea, with bacteria being dominant in terms of both abundance and function (Cross et al., 2018; Bi et al., 2023). Within the bacterial community, *Firmicutes* and *Bacteroidetes* were the two dominant phyla; the other phyla included *Actinobacteria*, *Proteobacteria*, *Verrucomicrobia*, and *Fusobacteria* (Hou K. et al., 2022).

Over billions of years of co-evolution, GM has come to be regarded as a “virtual organ” of the body that performs essential functions across multiple domains. These functions include participation in food digestion and nutrient absorption (Cai and Wei, 2023), regulation of the immune system (Ng et al., 2019), protection against pathogenic microbial colonization (Roth-Schulze et al., 2021), synthesis of vital amino acids and vitamins (Song et al., 2022), and metabolism of exogenous substances, including oral medications (Guan et al., 2022).

**FIGURE 3:**
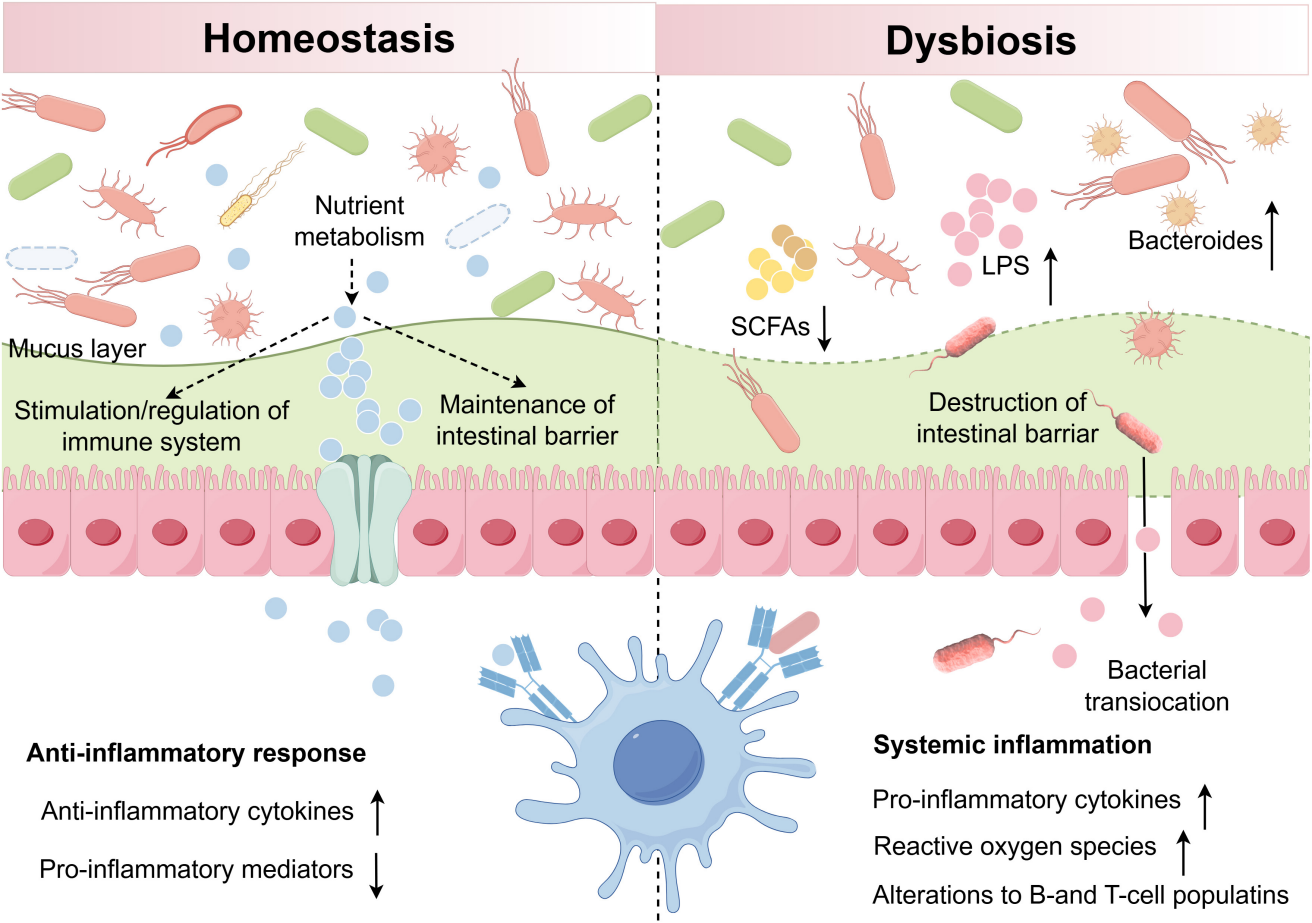
Intestinal microbiota in states of homeostasis and dysbiosis. Under homeostatic conditions (left), the GM supports nutrient metabolism, preserves the intestinal barrier integrity, and modulates immune activity to promote anti-inflammatory responses. Under dysbiotic conditions (right), alterations in microbial composition disrupt the barrier and elevate pro-inflammatory factors, leading to systemic inflammation. LPS, lipopolysaccharide, SCFAs, short-chain fatty acids.

Under conditions of homeostasis, the GM ferments dietary fiber to produce short-chain fatty acids (SCFAs) (Zhou et al., 2023), which play a crucial role in enhancing the integrity of the intestinal epithelial barrier, promoting immune tolerance, and regulating inflammatory responses through G protein-coupled receptors (Mehta et al., 2021; Bhargava et al., 2022). Notably, propionate and butyrate are essential for mitigating inflammation, such as uveitis, and maintaining intestinal homeostasis. Additionally, the GM synthesizes vital nutrients, including folate, vitamin K, and biotin, to support host health (Bäckhed et al., 2015). Conversely, dysbiosis leads to the proliferation of harmful pathogens, compromising the intestinal barrier and facilitating bacterial translocation. Pathogenic molecules, such as lipopolysaccharide (LPS), can enter systemic circulation, resulting in hyperactivation of the immune system, the release of pro-inflammatory cytokines and reactive oxygen species, and alterations in B and T cell populations (Wei et al., 2020; Figure 3).

## 4 Pathogenic mechanism of GM involvement in uveitis

**FIGURE 4:**
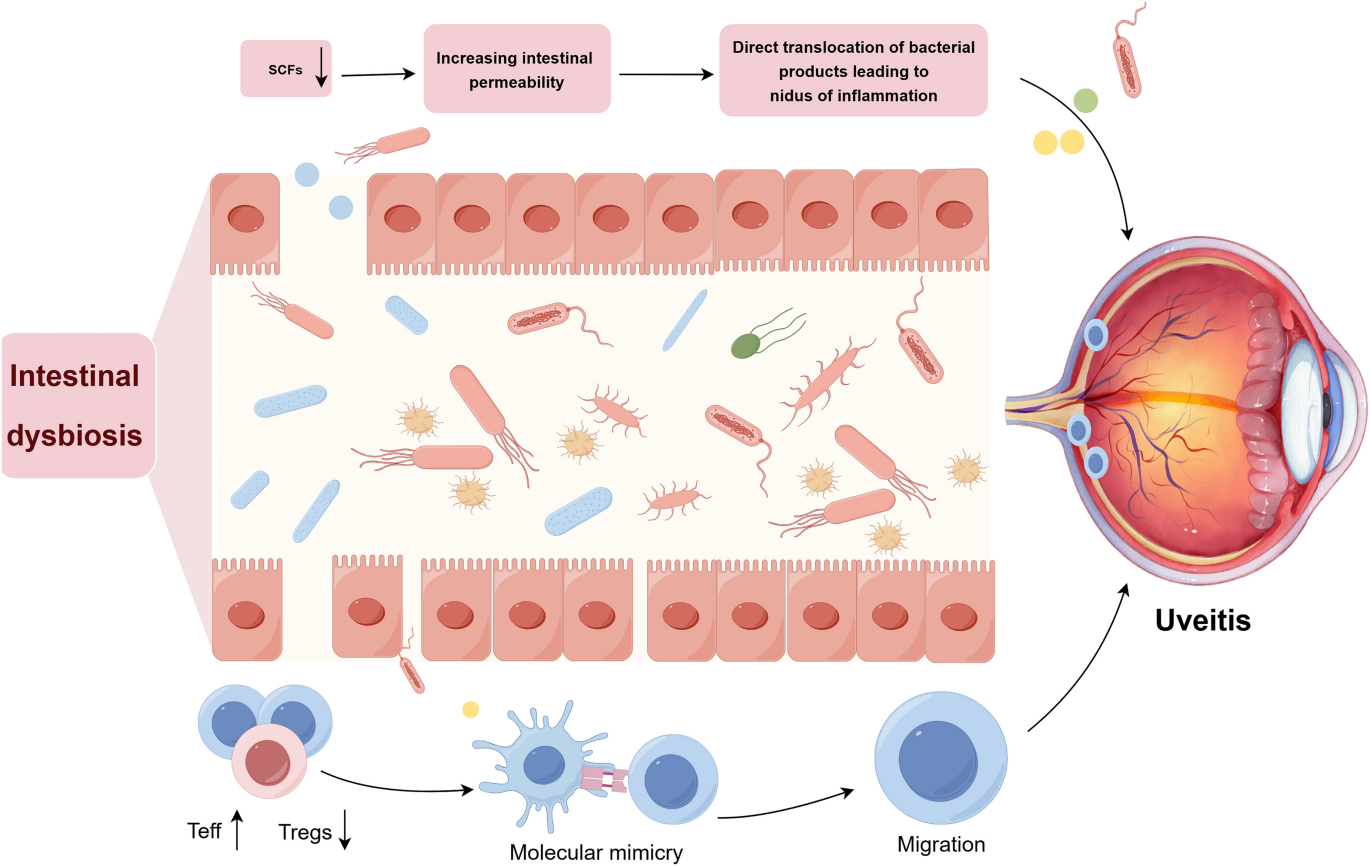
Mechanistic correlation between the gut microbiota and ocular autoimmunity. SCFAs, short-chain fatty acids; Teff, effector T cells; Treg, regulatory T cells.

Intestinal dysbiosis, a primary indicator of microecological imbalance within the gut, significantly modulates the ocular immune microenvironment via a complex network of molecular and immunological pathways. Dysbiosis is increasingly recognized as a pivotal factor in the initiation or exacerbation of uveitis. The mechanisms by which intestinal dysbiosis contributes to the onset and progression of uveitis are outlined below. Intestinal dysbiosis results in reduced production of SCFAs, resulting in two major alterations: compromised integrity of the intestinal barrier and disrupted balance of T-cell subsets, leading to an overabundance of effector T-cells and a deficiency of Tregs. The proliferation of effector T-cells stimulated by microbial antigens exhibiting molecular mimicry plays a critical role in uveitis pathogenesis. Furthermore, a compromised intestinal barrier facilitates the translocation of bacterial products to distal host tissues, including the ocular region, culminating in the development of inflammatory foci in these areas (Figure 4).

### 4.1 Molecular mimicry

**FIGURE 5:**
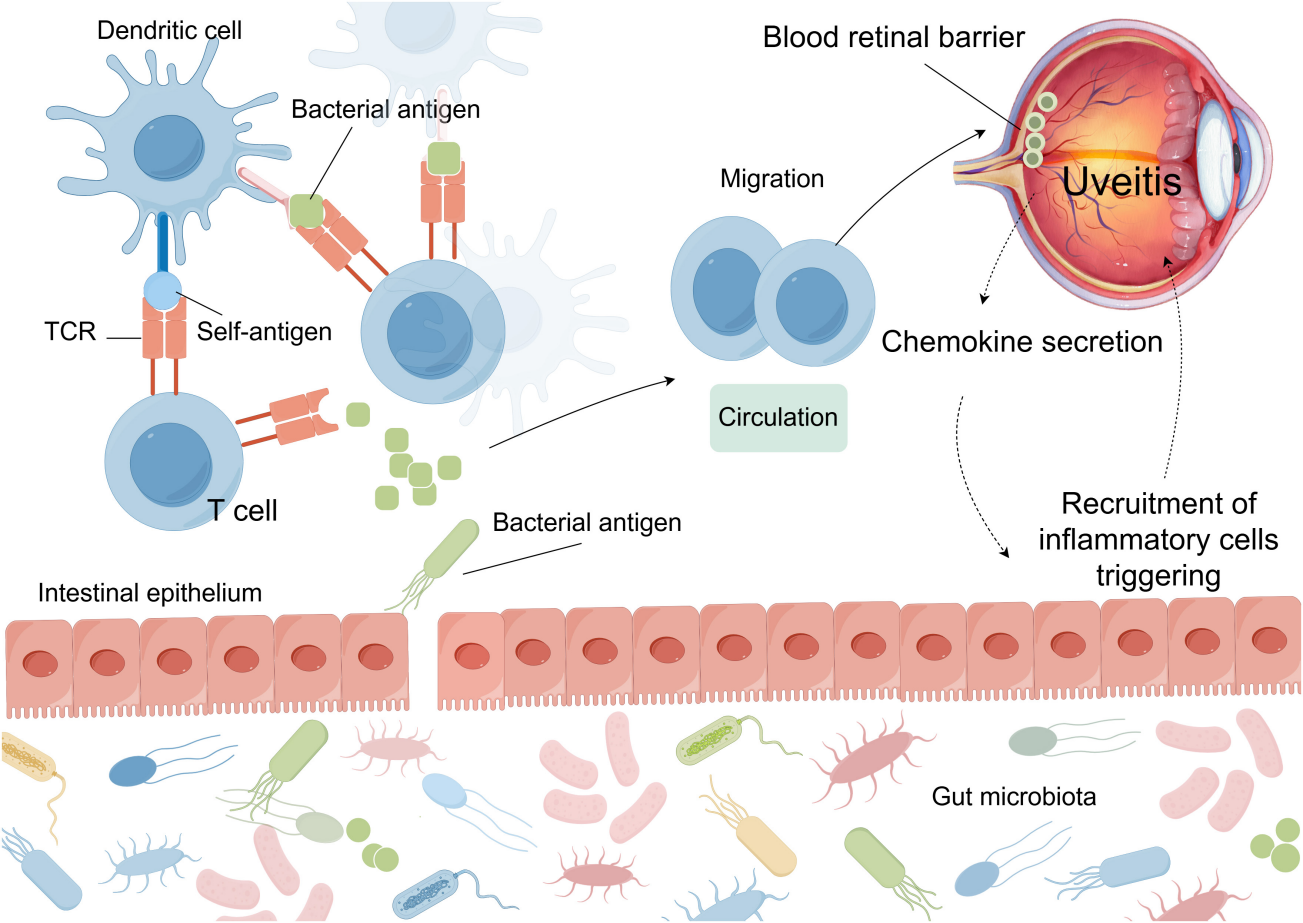
Molecular mimicry by microbial antigens activates autoreactive T cells, which then cross the blood-retinal barrier and initiate intraocular inflammation. TCR, T-cell receptor.

Molecular mimicry describes the phenomenon whereby GM imitates host antigen epitopes, leading to the activation of host-specific T-cells and consequently initiating an autoreactive T-cell immune response against self-antigens (Figure 5). From an evolutionary standpoint, gut microbes may enhance their survival within the host by mimicking host self-antigen structures, thereby facilitating immune evasion. This molecular-level structural similarity may result from homologous amino acid sequences, conserved nucleotide sequences, or analogous three-dimensional protein conformations (Rojas et al., 2018). These autoreactive T-cells can traverse the blood-retinal or blood-aqueous barrier, infiltrate the eye, and induce intraocular inflammation. An *in vitro* study exhibited that adoptive transfer of gut content-activated T-cells from mice with spontaneous EAU consequently induced uveitis in recipient mice. This evidence underscores the role of microbial antigen mimicry via gut contents in EAU pathogenesis (Horai et al., 2015).

A research team led by Rachel Caspi discovered that in a murine model of autoimmune uveitis, the disease could arise spontaneously without external immune stimulation. Notably, the use of antibiotics to deplete GM reduces both the incidence and severity of the disease. This finding implies that certain commensal gut bacteria may produce proteins structurally analogous to retinal antigens, such as interphotoreceptor retinoid-binding protein (IRBP), thereby activating pathogenic T-cells through molecular mimicry (Rojas et al., 2018). Additionally, antibodies against retinal S antigens have been associated with autoimmune uveitis (Wildner, 2023). Peptides derived from *Escherichia coli* share amino acid homology with retinal S-antigen peptides and can induce cross-reactive T-cell responses *in vitro*. Similarly, homologs of the retinal S-antigen from the hepatitis B virus, baboon endogenous virus, and rotavirus have been found to induce uveitis in Lewis rats following immunization (Singh et al., 1990). Although these studies provide experimental support for the antigen mimicry hypothesis, the specific microbial antigens that trigger this process in human uveitis remain unclear. Although these studies provide experimental support for the antigen mimicry hypothesis, the specific microbial antigens that trigger this process in human uveitis remain unclear. Consequently, identifying these triggers is critical for elucidating the precise mechanisms by which microbial antigens promote autoimmune uveitis.

### 4.2 Reduction of anti-inflammatory microbial metabolites

**FIGURE 6:**
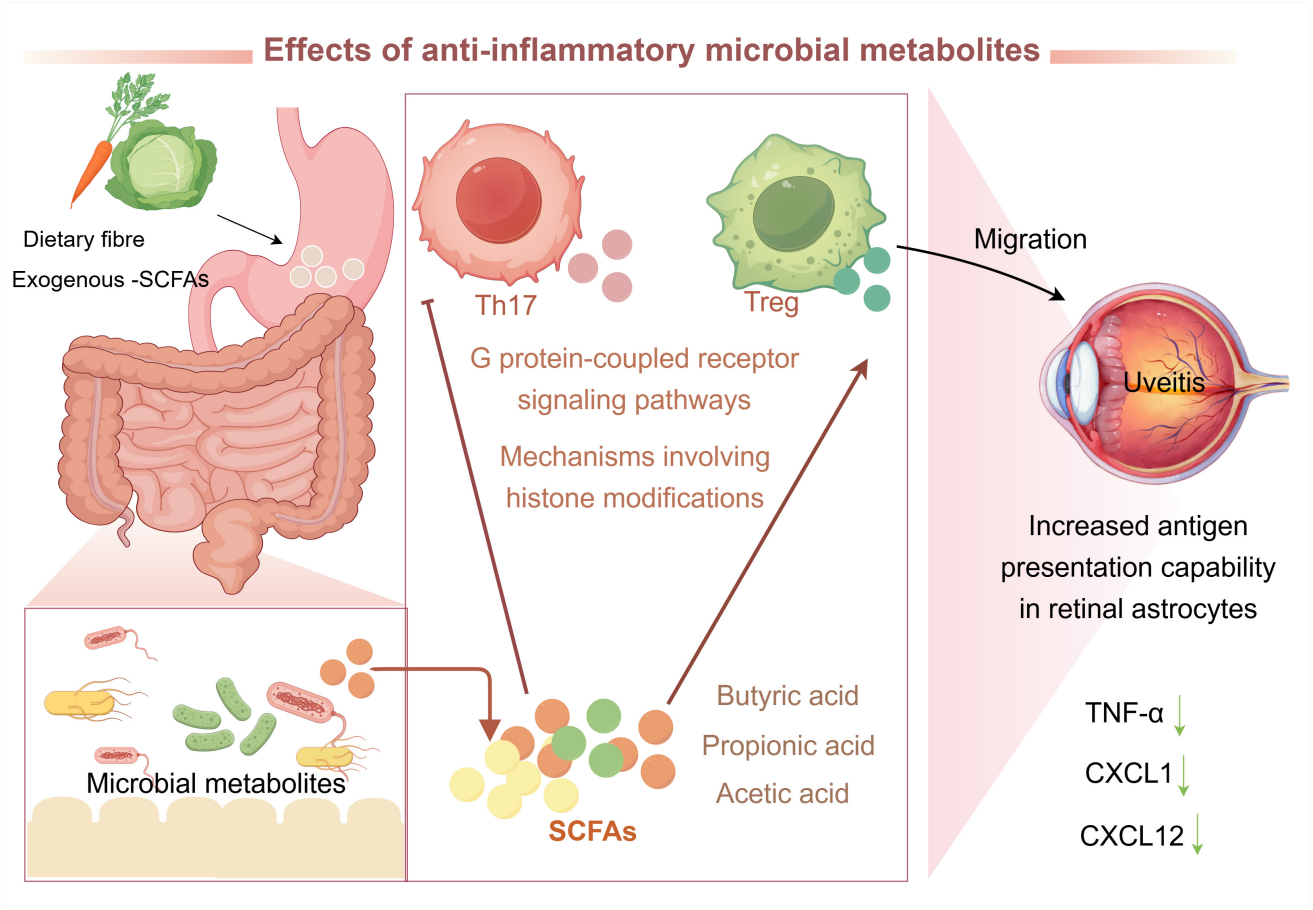
Gut microbiota (GM)-derived SCFAs enhance the integrity of the intestinal barrier, promote Treg-cell differentiation, and inhibit Th17-cell migration. SCFAs, short-chain fatty acids; Th17, T helper 17 cells; Treg, regulatory T cells; TNF-α, tumor necrosis factor-α; CXCL1, C-X-C motif chemokine ligand 1; CXCL12, C-X-C motif chemokine ligand 12.

Gut microbiota can produce a vast array of metabolites, including SCFAs, notably butyrate, propionate, and acetate, which are among the most prominent. These SCFAs are synthesized by GM through the fermentation of dietary fiber and are instrumental in maintaining host immune homeostasis (Zhang et al., 2020; Baccarelli et al., 2023). SCFAs predominantly facilitate the proliferation of Tregs in colonic lymph nodes and inhibit Th17 cell differentiation via mechanisms involving histone modifications and G-protein-coupled receptor signaling pathways. This activity is essential for preserving intestinal barrier integrity and modulating immune T cell responses (Figure 6; Sun et al., 2017).

Empirical studies have demonstrated that exogenous SCFA supplementation can mitigate uveitis in murine models. Specifically, in the C57BL/6J EAU model, oral administration of propionate induced Treg differentiation in the intestinal lamina propria and mesenteric lymph nodes, thereby reducing the severity of uveitis. Investigations utilizing Kaede transgenic mice to monitor cell migration have corroborated that during uveitis, there is increased migration of Th1 and Th17 cells between the distal colon and extra-intestinal lymphoid tissues. However, supplementation with SCFAs has been observed to diminish trafficking in both intestinal and extra-intestinal tissues (Nakamura et al., 2017). Moreover, intraperitoneally administered SCFAs can access the eye via the systemic circulation, thereby modulating the immune response of specific T-cells and promoting the proliferation of IRBP-specific T-cells. Additionally, SCFAs enhance the antigen-presenting capacity of retinal astrocytes and mitigate the production of cytokines and chemokines, such as TNF-α, C-X-C motif chemokine ligand 1, and C-X-C motif chemokine ligand 12, thereby alleviating LPS-induced uveitis (Chen N. et al., 2021). Consequently, exogenous SCFA supplementation has emerged as a promising therapeutic strategy for uveitis treatment. However, further studies are needed to validate their efficacy.

Short-chain fatty acids play a crucial role in regulating intestinal barrier function. Empirical studies conducted in rat models have demonstrated that acetate and propionate can effectively decrease duodenal permeability (Wan Saudi and Sjöblom, 2017). In addition to the role of SCFAs, fecal DNA analysis in patients with AAU has identified significantly elevated concentrations of seven specific metabolites, including 6-deoxy-D-glucose, linoleic acid, and N-acetyl-β-D-mannosamine, compared to healthy controls. Although these metabolites may exhibit pro-inflammatory properties, their precise mechanisms in the context of acute uveitis remain unclear (Huang et al., 2018).

In conclusion, gut dysbiosis leads to a reduction in SCFAs and alterations in the microbial community structure, which then increase intestinal permeability, promote excessive activation of effector T cells, and impair Treg function. Effector T-cells may be activated through molecular mimicry between microbial antigens and host ocular antigens, leading to an autoimmune attack on ocular tissues. Simultaneously, compromised intestinal barrier function allows the translocation of bacterial products to distant organs, including the eye, thereby initiating local inflammatory responses and ultimately contributing to the onset and progression of uveitis.

### 4.3 Increased intestinal permeability

**FIGURE 7:**
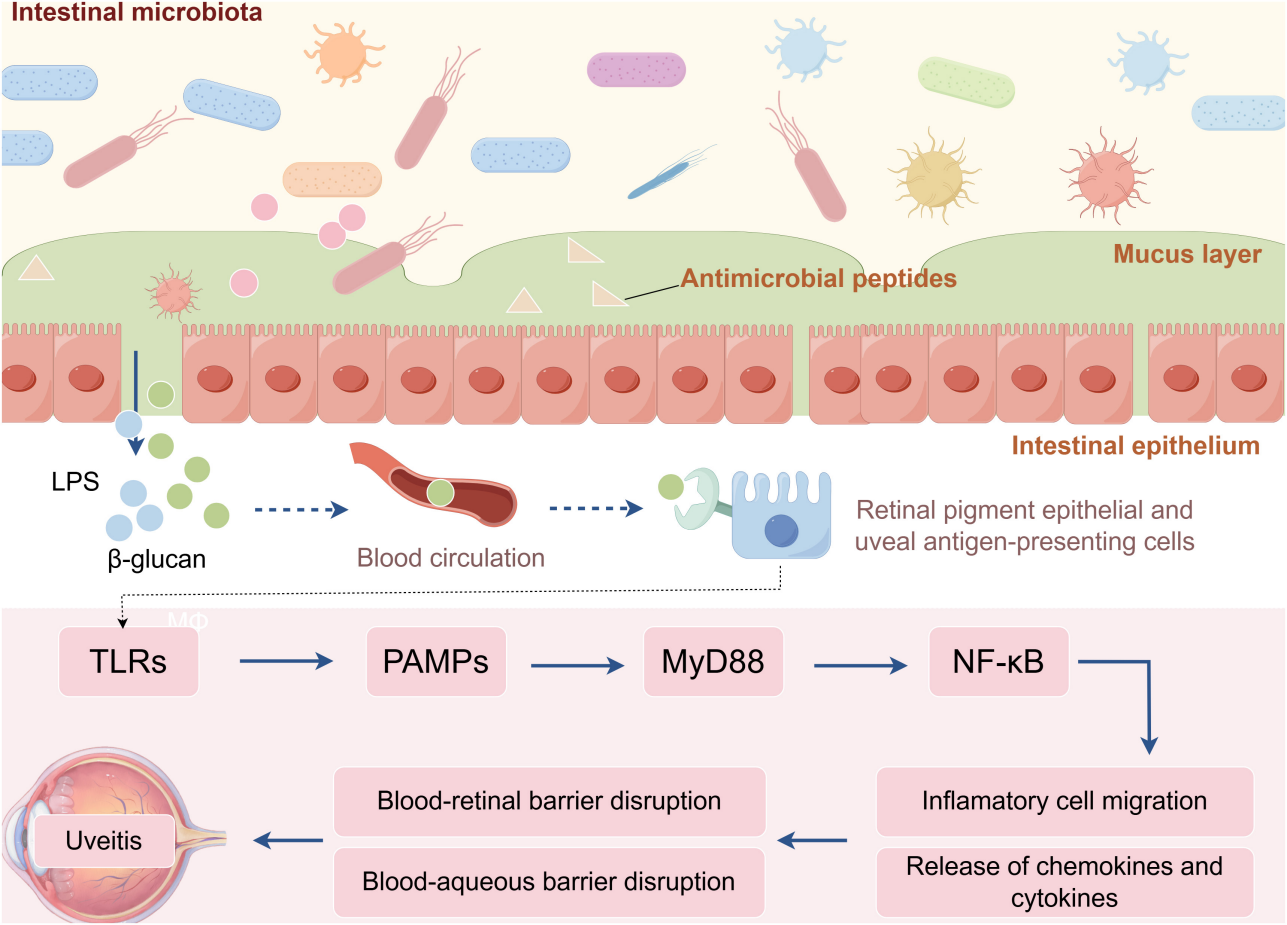
Mechanism of uveitis triggered by increased intestinal permeability. Gut dysbiosis disrupts the intestinal barrier, allowing microbes and their products to enter systemic circulation and migrate to the eye. In ocular tissues, TLRs on antigen-presenting cells recognize pathogen-associated molecular patterns (PAMPs) and activate the NF-κB pathway via MYD88. This activation induces the production of pro-inflammatory cytokines and chemokines, leading to disruption of the blood–retinal and blood-aqueous barriers and facilitating immune-cell infiltration, ultimately driving ocular inflammation. LPS, lipopolysaccharide; TLRs, toll-like receptors; PAMPs, pathogen-associated molecular patterns; NF-κB, nuclear factor kappa B; MYD88, myeloid differentiation factor 88.

The intestinal epithelium, mucus layer, and antimicrobial peptides are critical physical and chemical barriers to enteric pathogenic microorganisms (Bülck et al., 2023). Gut dysbiosis can induce mucosal inflammation and compromise this barrier, leading to increased intestinal permeability and facilitating the translocation of microbes and their metabolites, such as LPS and β-glucan, into the bloodstream, lymphatic system, or submucosal and lamina propria regions (Samalia et al., 2025). These microbial components can then be disseminated to the eye via the circulatory system, where they directly induce inflammatory responses in target organs (Janowitz et al., 2019). On the surface of antigen-presenting cells within the retinal pigment epithelium and uveal tract, toll-like receptors (TLRs) recognize pathogen-associated molecular patterns and activate nuclear factor kappa B (NF-κB) through the myeloid differentiation factor 88-dependent signaling pathway. This activation initiates downstream signaling cascades, promoting the release of various cytokines and chemokines that disrupt blood-retinal and blood-aqueous barrier structures. Consequently, this enhances immune cell migration into the eye, thereby driving the inflammatory processes (Figure 7; Li et al., 2020).

In the EAU mouse model, there was a positive correlation between increased intestinal permeability and both early onset and severity of the disease. Janowitz et al. (2019) demonstrated that in EAU mice immunized with IRBP peptides in conjunction with an inactivated *Mycobacterium tuberculosis* antigen adjuvant, there was a reduction in the expression of the tight junction protein zonula occludens-1, along with an increase in the transcellular permeability of fluorescently labeled dextran, indicating compromised intestinal barrier function. This increased permeability further aggravates uveitis progression. Furthermore, alterations in intestinal permeability were associated with significant changes in the microbial community structure. Linear discriminant analysis effect size analysis revealed that in comparison to the control group, mice with uveitis exhibited an increased abundance of *Clostridium* and *S24-7*, while the abundance of beneficial microbiota such as *Verrucomicrobia*, *Akkermansia*, and *Dorea* was reduced. Notably, during the peak phase of the disease, IRBP-immunized mice demonstrated a notable increase in genera, such as *Prevotella* and *Lactobacilli*, whereas the control group demonstrated greater enrichment of *Ruminococcus* and *Bacteroidia*. There is a significant correlation between the degree of intestinal inflammation and the severity of uveitis (Janowitz et al., 2019).

Similar observations have been reported in humans with AS-associated uveitis, where patients exhibited compromised gut-vascular barrier function, abnormal expression of tight junction proteins, and elevated serum LPS levels (Ciccia et al., 2017). Collectively, this evidence suggests that altered intestinal permeability may occur during the early stages of uveitis. As ocular inflammation progresses, the degree of permeability increases further, interacting with pre-existing dysbiosis to form a critical link that drives disease progression.

### 4.4 Loss of intestinal immune homeostasis

**FIGURE 8:**
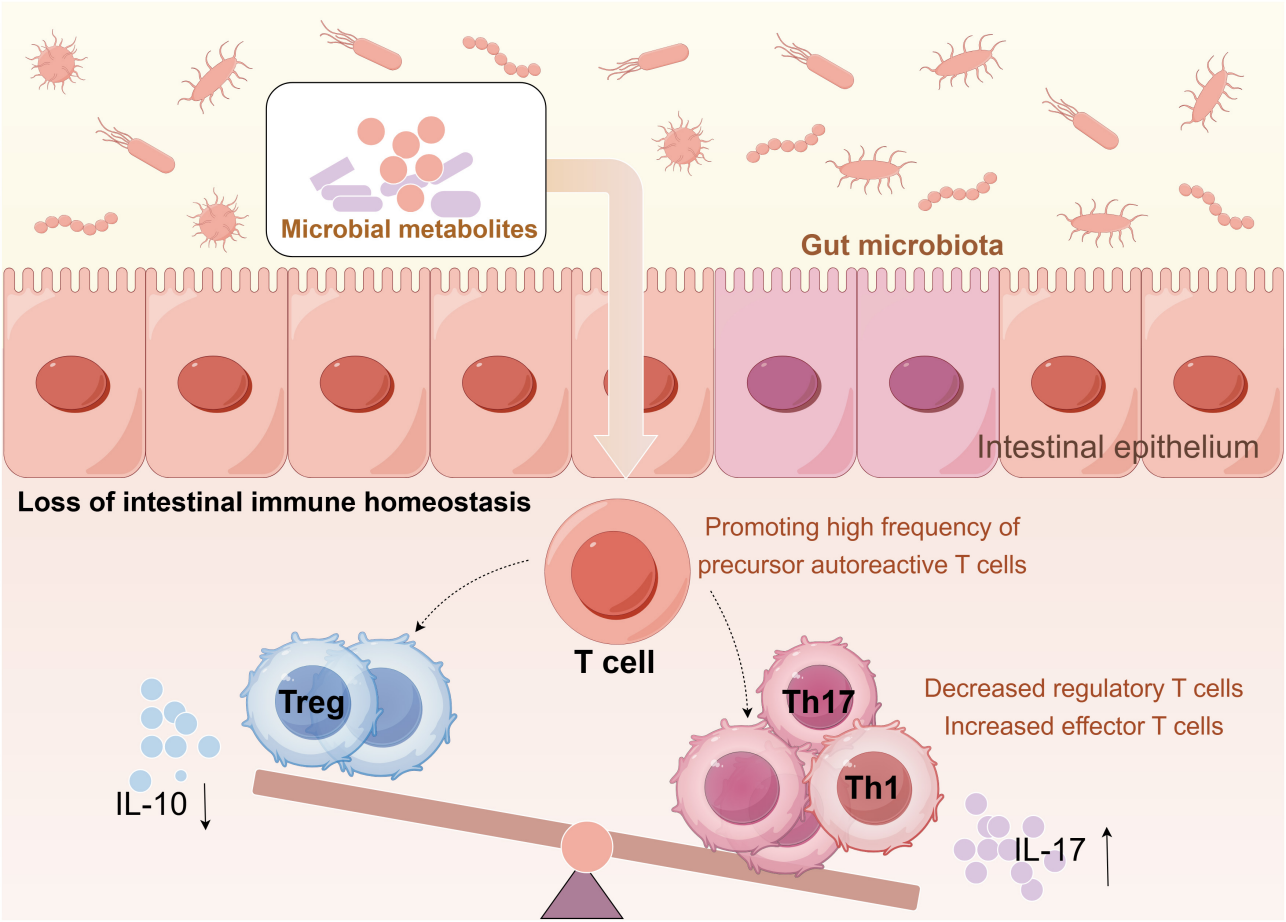
Loss of intestinal immune balance. Gut dysbiosis leads to an increase in pro-inflammatory effector T-cells and a decrease in Tregs, thereby disrupting intestinal immune homeostasis. Treg, regulatory T cell; Th17, T helper 17 cell; Th1, T helper 1 cell; IL-10, interleukin-10; IL-17, interleukin-17.

T cells play a central role in the pathogenesis of autoimmune diseases. Under normal immune conditions, a dynamic equilibrium is maintained between pro-inflammatory effector T-cells, such as Th1 and Th17 cells, and Tregs, which regulate the intestinal immune homeostasis. Within the gut-associated lymphoid tissue, antigen-presenting cells and associated helper T-cells identify and present pathogenic antigens, thereby stimulating the proliferation of Th1 and Th17 cells and initiating an inflammatory response. Conversely, Tregs perform immunosuppressive functions that prevent excessive immune activation and chronic inflammation. The equilibrium between these two T-cell subsets is crucial for sustaining intestinal immune homeostasis (Omenetti and Pizarro, 2015). However, homeostasis is disrupted during dysbiosis, resulting in an imbalance in the Th17/Treg ratio. This imbalance is characterized by an increase in Th17 cells and their effector cytokine IL-17, along with a decrease in Tregs and the anti-inflammatory mediator IL-10, collectively fostering aberrant immune activation and inflammatory responses (Figure 8; Gao et al., 2025; Zhuang et al., 2017). This mechanism was demonstrated in a murine EAU model. Empirical evidence indicates that broad-spectrum oral antibiotics modify GM composition, specifically by decreasing the abundance of *Firmicutes*, *Bacteroidetes*, and *Alphaproteobacteria*, while increasing *Gammaproteobacteria*, and significantly mitigating the severity of uveitis by enhancing the proportion of Tregs in various lymphoid tissues and the ocular region (Nakamura et al., 2016).

Current understanding suggests that innate immune molecules activated by GM constitute a critical source of cross-reactive antigens in the eye. Under normal physiological conditions, ocular antigens do not provoke pathological immune responses owing to the immune privilege of the eye; however, during intestinal dysbiosis, molecules that are structurally analogous to retinal antigens may be synthesized. These molecules activate retina-specific T-cells in the intestinal lamina propria, enabling them to cross the blood-retinal barrier and differentiate into Th17 and Th1 cells, both of which are key effector cells in uveitis pathogenesis (Horai et al., 2015). Furthermore, Nakamura et al. (2016) observed an increase in gut bacteria of the genera *Clostridium*, *Coprococcus*, and *Dorea*, along with a decrease in *Ruminococcus* and *Oscillospira*, in experimental EAU mice. This dysbiotic state is associated with enhanced lymphocyte activation in the gut, characterized by an increase in Th17 cells and a reduction in Tregs. Thymus-derived Tregs negatively regulate effector immune cells to maintain intestinal immune homeostasis. Studies in EAU mice have confirmed that oral antibiotic treatment increases Treg populations in the intestinal lamina propria and extra-intestinal lymphoid tissues while reducing effector T-cells. This shift correlates with attenuated inflammatory vascular damage in the retina and reduced uveitis severity, suggesting a mechanism mediated by GM modulation.

## 5 Gut-eye axis

Since Rowan et al. (2017) introduced the concept of the “gut-retina axis” in AMD research in 2017, the role of GM as a potential modulator of ocular diseases has received considerable scholarly attention, prompting extensive investigation. Current research has unequivocally demonstrated that key factors, including dietary habits, lifestyle choices, and medication use, can indirectly influence the onset and progression of ocular diseases by modifying GM composition and function (Bringer et al., 2022). Studies have further corroborated that dysbiosis, or disruption of GM homeostasis, is closely associated with the pathological mechanisms underlying various ocular conditions.

This influence encompasses prevalent eye diseases, such as uveitis, dry eye syndrome, AMD, diabetic retinopathy, and glaucoma (Wang and Sun, 2022). Consequently, GM has emerged as a central focus for demonstrating the pathogenesis of ocular disorders and developing intervention strategies. In a bibliometric analysis of the correlation between GM and ocular diseases, Fu et al. (2023) identified 284 relevant publications in this domain, spanning from 2009 to 2023. A notable increase in publication frequency has been observed since 2016, reflecting escalating research interest in this field. The literature further substantiates the concept of a “gut-eye axis,” wherein gut dysbiosis may play a pivotal role in the initiation and progression of various ocular diseases. Mechanistically, GM exhibits metabolic activity similar to that of other human organs, producing a diverse array of bioactive compounds, such as SCFAs, bacteriocins, secondary bile acids, indole derivatives, and polyamines via intricate metabolic pathways (Xue et al., 2021). These metabolites can be transported via systemic circulation to ocular tissues, where they may directly or indirectly modulate physiological functions and contribute to the pathogenesis of ocular diseases, thereby serving as potential mediators of disease development (Napolitano et al., 2021b; Zhang et al., 2023). The “gut-eye axis” hypothesis posits that an imbalance in the GM or a compromised intestinal barrier may facilitate the translocation of gut microbes and their metabolites, consequently influencing distant ocular tissues and contributing to the pathogenesis of ocular disorders (Moon et al., 2020).

**FIGURE 9:**
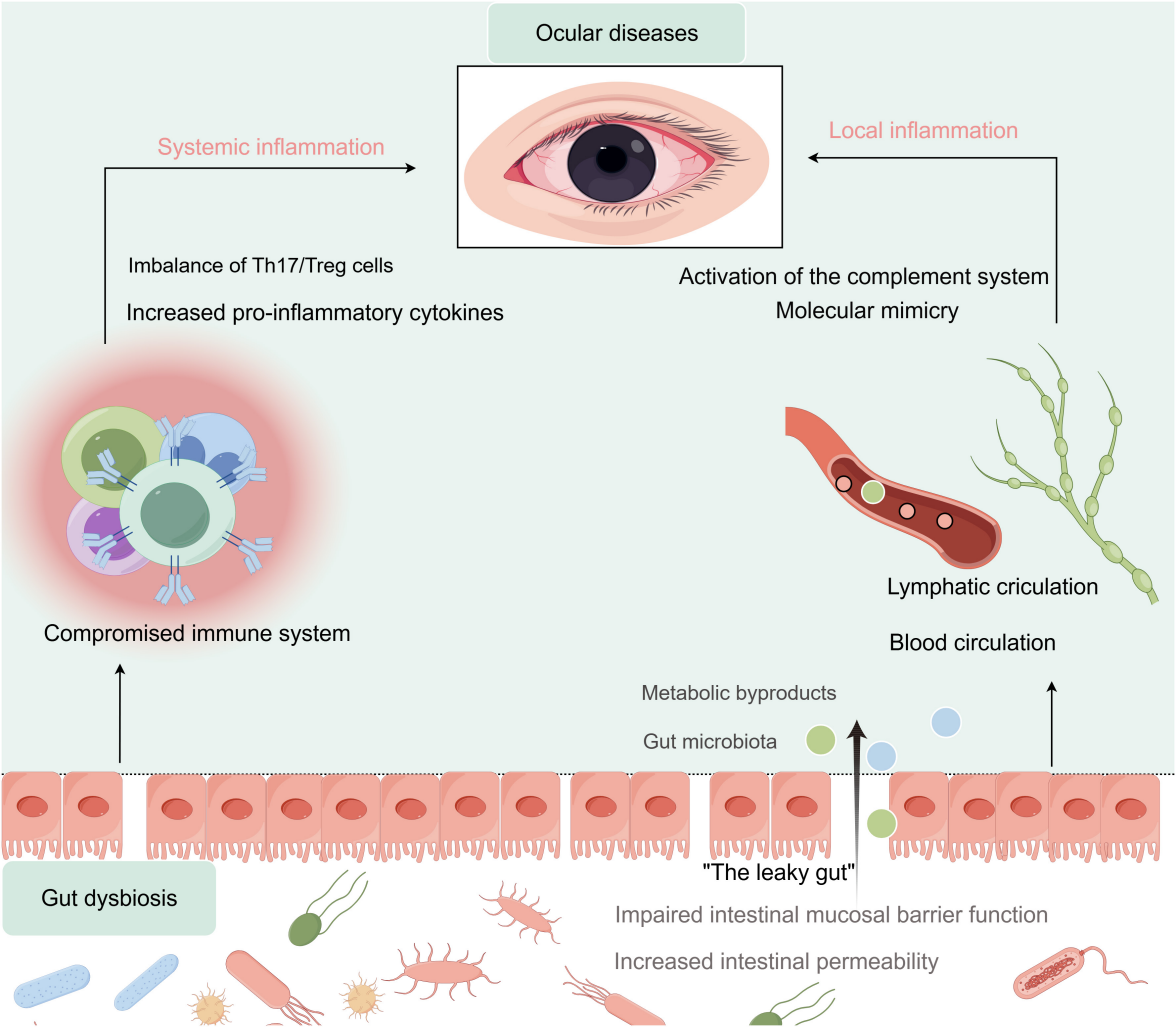
The gut-eye axis. Schematic illustration of the two mechanistic pathways underlying the connection between the gut microbiota and ocular inflammation.

Currently, the academic community has identified two potential mechanistic frameworks for demonstrating the relationship between GM and ocular pathologies (Figure 9). The first mechanism centers on “immune dysregulation and systemic inflammation.” The second mechanism, closely related to the previously discussed process, is “gut barrier disruption and microbial translocation.” This involves immune dysregulation induced by gut dysbiosis, which can lead to compromised intestinal mucosal barrier function and increased intestinal permeability, a pathological condition commonly referred to as “leaky gut” (Camilleri, 2019; Camilleri and Vella, 2022).

In summary, the two mechanisms constitute a synergistic pathogenic cascade in which gut microbiota dysbiosis precipitates immune dysregulation, systemic inflammation, and compromise of the intestinal barrier, ultimately resulting in microbial translocation and perpetuating a vicious cycle. This dual-mechanism framework reinterprets ocular pathogenesis through the lens of the gut-eye axis, proposing extraocular origins and endorsing targeted adjunctive interventions. Future research should aim to elucidate the specific microbial taxa involved, the molecular interactions at play, and the effectiveness of gut-targeted strategies to enhance personalized care.

## 6 Gut microbiome in systemic diseases associated with uveitis

Autoimmune uveitis is frequently associated with a range of systemic conditions, including Behçet’s disease, Vogt-Koyanagi-Harada disease, HLA-B27-associated uveitis, systemic sarcoidosis, ankylosing spondylitis (AS), multiple sclerosis (MS), and inflammatory bowel disease (IBD). A growing body of evidence suggests the presence of gut microbial dysbiosis in patients with systemic disorders. From a broader perspective, if microbiome signatures specific to uveitis subtypes can be identified, population-level screening could potentially help detect individuals at an elevated risk of developing uveitis.

### 6.1 Behçet’s disease (BD)

Behçet’s disease is a systemic autoinflammatory condition predominantly characterized by recurrent uveitis, with potential involvement of the mucosal, cutaneous, ocular, vascular, nervous, and gastrointestinal systems (Zhang et al., 2018; Hou C. et al., 2022). Recent research has identified significant alterations in the composition of the GM in patients with BD compared to that in healthy individuals (Yasar Bilge et al., 2020). Specifically, patients with BD demonstrate an increased relative abundance of *Firmicutes*, *Clostridia*, *Clostridiales*, and *Prevotella copri* within their fecal microbiota (Tecer et al., 2020), whereas the gut microbiome of healthy individuals is primarily dominated by *Bacteroidetes* (Mizuki et al., 2010). In a metagenomic analysis of fecal samples from 22 patients with BD, Consolandi et al. (2015) reported a reduction in microbial diversity and a marked decrease in butyrate-producing bacteria. Butyrate, an essential microbial metabolite, is reduced in patients with BD, which may lead to compromised Treg function and enhance pathogenic Th1/Th17 immune responses, thereby exacerbating inflammation (Emmi et al., 2021; Mehmood et al., 2021). Conversely, *Bacteroidetes*, which are abundant in healthy GM, produce SCFAs, including butyrate, that help maintain immune homeostasis. Functional experiments have further demonstrated that fecal microbiota transplantation (FMT) from patients with Behçet’s active disease into experimental autoimmune uveitis (EAU) mouse models aggravates ocular pathology, accompanied by impaired intestinal barrier integrity and increased expression of the pro-inflammatory cytokines interferon-γ (IFN-γ) and interleukin-17 (IL-17) (Wang et al., 2021). These findings indicated that GM dysbiosis in BD may contribute to disease pathogenesis by modulating the host immune response.

Collectively, these studies establish a connection between gut microbiota dysbiosis and BD, particularly concerning its ocular manifestations. The observed microbial alterations, characterized by an increase in Firmicutes and a decrease in Bacteroidetes and butyrate-producing bacteria, along with disruptions in SCFA metabolism and the Treg/Th17 balance, suggest a potential mechanism by which gut dysbiosis may drive inflammation in BD. FMT experiments further substantiate this association, linking BD-related microbiota with exacerbated ocular pathology and compromised intestinal barrier function. These findings contribute to the understanding of the gut-eye axis; however, they underscore the necessity for additional research to confirm causality, identify biomarkers, and investigate gut-targeted therapeutic strategies, such as probiotics and FMT.

### 6.2 Vogt-Koyanagi Harada (VKH) disease

Vogt-Koyanagi Harada disease is an autoimmune condition primarily characterized by bilateral granulomatous panuveitis (Yang et al., 2018). Emerging research indicates that the GM may play a significant role in its pathogenesis. Patients with active VKH disease frequently demonstrate a reduction in butyrate-producing bacteria, lactate-producing bacteria, and methanogens, along with a relative increase in gram-negative pathogenic bacteria, such as those of the genus *Prevotella*. Notably, immunosuppressive treatment has been observed to partially restore GM composition in patients with VKH disease. Animal models have further demonstrated that transplantation of GM from patients with VKH disease significantly exacerbates disease severity in EAU mice (Ye et al., 2020). Microbiome analyses of fecal samples from untreated patients with active VKH disease revealed significant differences in GM composition compared to healthy controls. Li M. et al. (2022) reported that patients with VKH disease exhibited higher relative abundances of *Stomatobaculum*, *Pseudomonas*, and *Lachnoanaerobaculum*, whereas those of *Gordonibacter* and *Slackia* were significantly lower. Another study by Li et al. (2025) employed an integrative approach combining 16S rDNA sequencing with liquid chromatography-tandem mass spectrometry to compare the gut microbiome and its metabolites between patients with active VKH disease and healthy controls. These findings revealed that patients with VKH disease displayed distinct dysbiosis and metabolic disturbances. Notably, six microbial genera, *Pediococcus*, *Pseudomonas*, *Rhodococcus*, *Photobacterium*, *Gardnerella*, and *Lawsonia*, and two metabolites, pyrrolidine and epigallocatechin, were identified as potential biomarkers for differentiating VKH disease, with area under the curve values exceeding 0.82. In conclusion, current evidence suggests that patients with VKH disease exhibit structural abnormalities in GM and altered functional metabolites. These specifically altered microbial communities may serve as microbial biomarkers for VKH disease, with potential applications in auxiliary diagnosis, treatment guidance, and the prediction of therapeutic efficacy.

### 6.3 HLA-B27-associated uveitis

A significant association was observed between HLA-B27 expression and the development of uveitis. Approximately one-third of patients with HLA-B27-positive AS develop acute anterior uveitis (AAU), the pathogenesis of which may involve mechanisms such as HLA-B27-dependent gut dysbiosis, altered intestinal permeability, and molecular mimicry (Parthasarathy et al., 2023). Although comparative studies of fecal samples from patients with AAU and healthy controls have not revealed significant differences in GM composition, distinct variations have been observed at the microbial metabolic phenotype level (Huang et al., 2018). This observation suggests that the genotype may primarily influence disease involvement through modulation of microbial function rather than structural composition. Other HLA-associated diseases, such as celiac disease and the HLA-DQ2 genotype, have been found to alter the infant gut microbial composition, thereby increasing susceptibility to disease (Olivares et al., 2015). Furthermore, studies have demonstrated that microbial peptides may bind to HLA-B27 molecules, initiating specific immune responses in target organs such as the eyes and joints (Bodis et al., 2018). HLA-B27 significantly influences the intestinal microenvironment. Prior to the onset of dysbiosis in animal models, colonic antimicrobial peptides, including regenerating islet-derived protein III and S100 calcium-binding protein A8 (S100A8), are upregulated (Asquith et al., 2016). Notably, S100A8, a calcium-binding protein, is elevated in cases of endogenous posterior uveitis, and serum calprotectin levels have been suggested as potential biomarkers for uveitis associated with juvenile idiopathic arthritis (Walscheid et al., 2015).

These findings highlight the intricate crosstalk among the HLA-B27 genotype, gut microbial function, and ocular autoimmunity. They provide a novel perspective that targeting microbial functional pathways may yield more favorable therapeutic effects than merely modulating gut microbiota structure in cases of HLA-associated uveitis. Biomarkers including S100A8 and serum calprotectin hold considerable promise for the early diagnosis and dynamic monitoring of uveitis subtypes. Nevertheless, the precise molecular mechanisms underlying HLA-B27-mediated regulation of microbial metabolism and subsequent induction of ocular immune responses remain to be fully elucidated, and the specificity of these candidate biomarkers across distinct uveitis subtypes warrants further validation. Future investigations should focus on deciphering this molecular regulatory network and exploring targeted intervention strategies, such as microbial metabolite modulation, to optimize the clinical management of HLA-associated uveitis.

### 6.4 Ankylosing spondylitis

Although significant differences in the gut microbiome have been observed between patients with AS and healthy controls, a specific or consistent microbial signature for AS has not yet been established (Harkins et al., 2021). Notably, studies, particularly those involving Chinese cohorts, have reported a reduction in *Bacteroides*, whereas an Italian study reported decreased levels of *Veillonellaceae* and *Prevotellaceae* in patients with AS. Morandi et al. (2024) identified that patients with HLA-B27-associated uveitis exhibited elevated levels of *Eubacterium* and an upregulation of lipid biosynthesis pathways compared to controls. These findings further support an association between microbial dysbiosis and uveitis. More recently, a large-scale study utilized Mendelian randomization to investigate the causal relationship between specific GM and the risk of developing AS. *Bacteroides vulgatus* was associated with an increased risk (odds ratio 1.55); 11 bacterial traits were also associated with a heightened risk, and three traits indicated a reduced risk (Jiang et al., 2024).

### 6.5 Systemic sarcoidosis

Sarcoidosis is a multisystem granulomatous disease of unknown etiology that can affect virtually all organ systems, with approximately 30%–50% of patients developing associated uveitis (Lapa et al., 2018). In the context of microbial associations, *Mycobacterium* species and *Cutibacterium acnes* are identified as the most relevant microorganisms associated with sarcoidosis (Fang et al., 2016; Nagata et al., 2017). Some studies have hypothesized that mycobacteria may contribute to the pathogenesis of the disease by eliciting a type IV hypersensitivity reaction, wherein T cells initiate an immune response against *M. tuberculosis*-specific antigens. In a study conducted by Nagata et al. (2017), retinal biopsy samples from 11 patients with sarcoidosis revealed positive staining for *Cutibacterium acnes* in nine cases (82%), whereas no such detection was observed in the control group with non-sarcoid uveitis. This observation suggests a potential role for *C. acnes* in the pathogenesis of ocular sarcoidosis, although further research is necessary to demonstrate its precise mechanisms.

### 6.6 Multiple sclerosis

A study conducted by the International MS Microbiome Study revealed that the composition of the GM in patients with untreated MS differs significantly from that in healthy individuals. Notably, there was a marked increase in the abundance of *Akkermansia muciniphila, Ruthenibacterium lactatiformans*, *Hungatella hathewayi*, and *Eisenbergiella tayi*, whereas the abundance of *F. prausnitzii* and *Blautia* species was significantly decreased. Additionally, untreated patients with MS demonstrated overexpression of the phytate degradation pathway, coupled with a notable reduction in carbohydrate metabolic pathways that produce pyruvate. Variations in microbial community structure have also been observed in patients with MS undergoing different pharmacological treatments (iMSMS Consortium, 2022). These findings suggest a correlation between specific gut microbiome profiles and the risk of MS, disease progression, and functional microbial changes in response to treatment. In cases of MS-associated anterior or intermediate uveitis, similar GM dysbiosis has been identified, characterized by a reduction in *Clostridia* clusters IV and XIVa, which are recognized for their roles in promoting Treg differentiation (Miyake et al., 2015). However, current research on MS-associated uveitis is limited. Moreover, given that much of the evidence linking GM with MS is primarily derived from animal studies, a definitive connection and its underlying mechanisms require further confirmation.

### 6.7 Inflammatory bowel disease

Clinical investigations have demonstrated notable alterations in the intestinal microbiota composition of IBD patients. Compared to healthy individuals, there is a significant reduction in the abundance of beneficial bacteria such as *F. prausnitzii* and *Roseburia intestinalis* in patients with Crohn’s disease (CD) and/or ulcerative colitis (UC). Conversely, there is an increased relative abundance and proliferation capacity of bacteria such as *Gamma-proteobacteria* and *Enterobacteriaceae*, including *E. coli* (Wu et al., 2023; Foppa et al., 2024). This perspective is corroborated by a study conducted by Guo et al. (2021), which assessed the presence of *F. prausnitzii* and *E. coli* among 28 healthy individuals, 45 patients with CD, 28 patients with UC, and 10 patients with irritable bowel syndrome. The findings confirmed that *F. prausnitzii* serves as a specific biomarker for IBD, as its abundance in IBD patients was significantly lower compared to IBS patients and healthy controls. Further analysis of the intestinal microbiota in IBD patients with varying levels of disease activity revealed a significant increase in the abundances of *Proteobacteria* and *Enterococcaceae*, alongside a notable decrease in the abundances of *Ruminococcaceae* and *Clostridiales* in patients with moderate to severe CD. It is noteworthy that the majority of these differences in intestinal microbiota associated with disease activity are linked to *Firmicutes*, *Bacteroidetes*, and *Proteobacteria* (Zhou et al., 2018).

## 7 Gut microbiome in infectious pathogens associated with uveitis

Building on the established role of gut dysbiosis in autoimmune uveitis, recent evidence indicates that an imbalance in intestinal microecology also significantly influences the onset and progression of infectious uveitis. Infectious uveitis is an ocular inflammatory condition directly induced by various pathogens, including *M. tuberculosis* and *Toxoplasma gondii* (Méndez-Rodríguez et al., 2025). Although there is currently no direct evidence of specific alterations in the gut microbiota of human patients with infectious uveitis, studies using animal models suggest that gut dysbiosis may serve as a critical “amplifier” and “facilitator” of intraocular inflammation in the context of infection. In a mouse model of *T.* gondii infection, it was found that the infection not only systemic pathologies but also significantly diminished gut microbial diversity (Chen et al., 2024). Notably, there was a reduction in *Bacteroidetes*, an increase in *Lactobacillus*, and an altered *Firmicutes/Bacteroidetes* ratio. The microbial disturbance coincided with immune dysregulation, characterized by hyperactivation of the Th1/Th17 cell pathways. This hyperactivation led to an increased secretion of the pro-inflammatory cytokines IFN-γ and IL-17, while the function of Tregs, which are essential for suppressing inflammation, was compromised. Consequently, this imbalance resulted in uncontrolled inflammatory responses. Similarly, in the EAU model, the use of mycobacterial antigens as an adjuvant to induce the disease was associated with significant alterations in gut microbiota (Janowitz et al., 2019). These alterations were marked by an increase in *Firmicutes* and a decrease in *Bacteroidetes*. Such changes indicate that the systemic immune response elicited by pathogenic infection or its antigens can remotely disrupt the stability of the gut microenvironment. The dysregulated gut microbiota and its metabolites may, in turn, influence systemic immune status through the “gut-eye axis,” potentially exacerbating immune-mediated attacks on ocular tissues or contributing to inflammatory damage. In summary, within the pathological context of infectious uveitis, pathogens are the primary initiators of inflammation. However, preliminary evidence from animal studies suggests that gut dysbiosis may act as a regulatory interface, potentially affecting the progression and severity of the inflammatory response.

## 8 Modulating GM to treat uveitis

**FIGURE 10:**
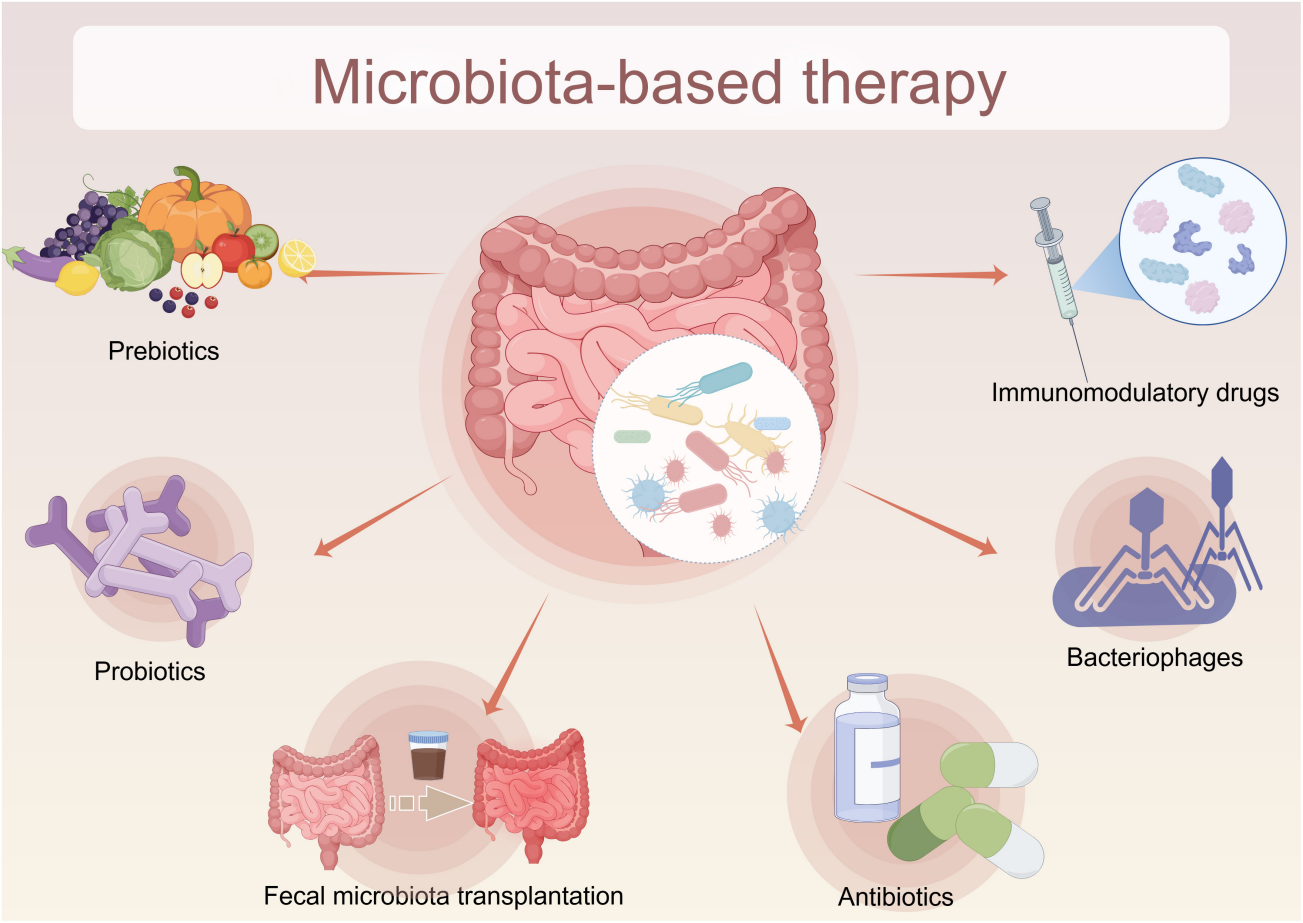
Therapeutic approaches for uveitis through gut microbiome modulation.

Targeted modulation of the gut microbiome is a promising therapeutic strategy for uveitis treatment. This approach can be implemented through various methodologies, such as administering probiotics, prebiotics, antibiotics, immunosuppressants, phage therapy, and FMT (Figure 10). Notably, probiotics and prebiotics are frequently combined with dietary interventions to restore gut microbial equilibrium (Ren et al., 2021). Probiotics rectify microbial dysbiosis and enhance intestinal barrier integrity, thereby reducing systemic inflammation (Jukic et al., 2021). Prebiotics, as dietary fibers, provide substrates for microbial fermentation, producing SCFAs that facilitate the differentiation and function of Tregs (Sima et al., 2018). Conversely, antibiotic therapy primarily aims to diminish systemic inflammation by eradicating pathogenic pro-inflammatory bacteria (Afsharipour et al., 2022). Phage therapy offers a more targeted approach, utilizing specific bacteriophages to lyse pathogenic bacteria while largely preserving the commensal microbiota (Lee et al., 2024). FMT provides a direct means of microbial restoration by introducing a diverse and stable microbial community into the gut, which may help modulate host immune responses and potentially reduce disease severity (Merrick et al., 2025). Collectively, these interventions seek to transform a dysbiotic gut ecosystem into a state that favors immune tolerance, thereby opening new avenues for the clinical management of uveitis.

### 8.1 Probiotics

**FIGURE 11:**
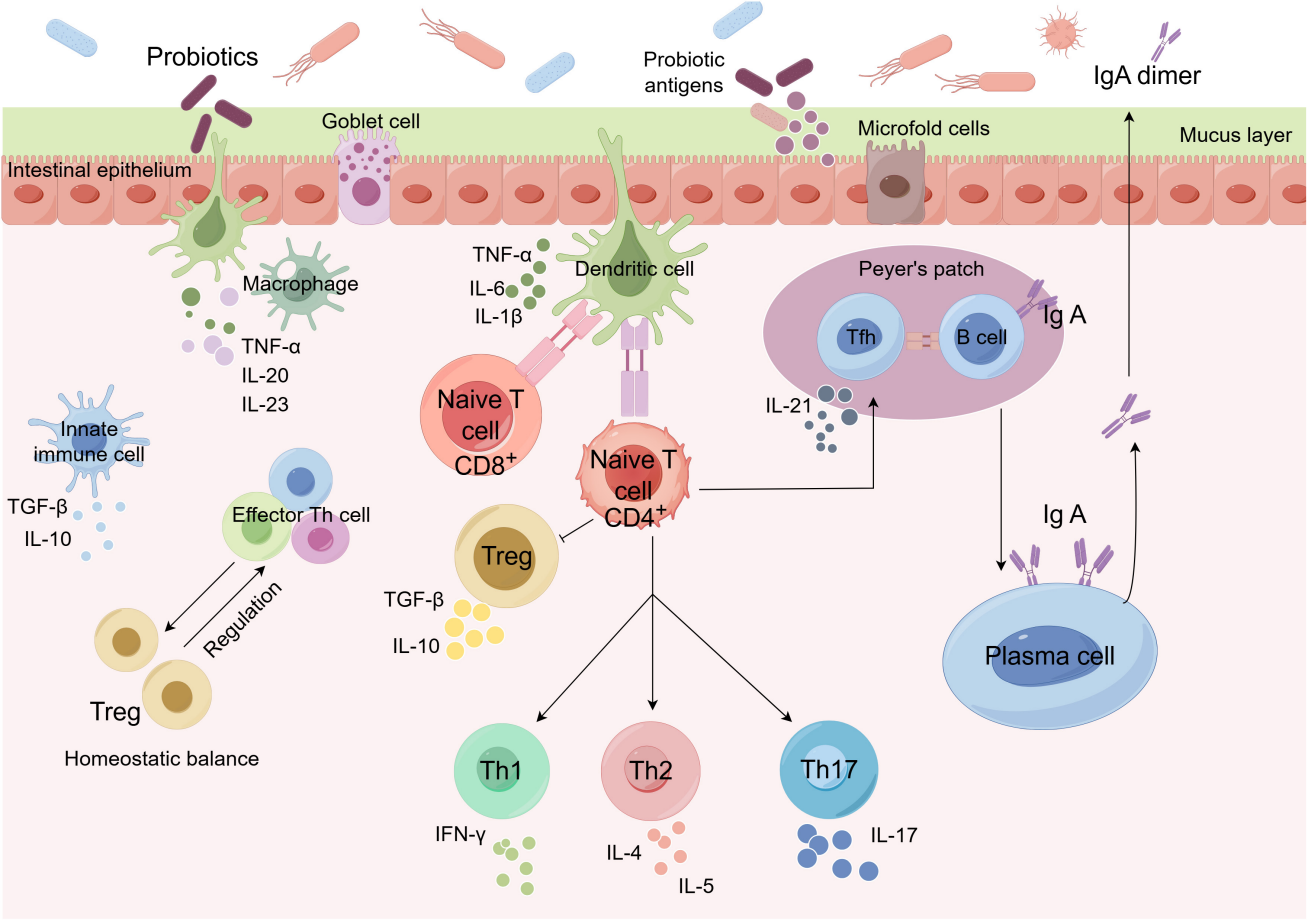
Schematic diagram of the interaction between host intestinal immune cells and probiotics. Probiotics play a role in immune responses by modulating immune cells such as dendritic cells, macrophages, and B and T lymphocytes. The goblet cells secrete a mucus layer that separates the GM from the intestinal epithelium. Ingested probiotics adhere to intestinal epithelial cells and activate them through pattern recognition receptors. Cytokines produced in response to probiotic stimulation activate Tregs, thereby maintaining the intestinal mucosal immune homeostasis. Intestinal antigens are delivered to dendritic cells via specialized intestinal cells known as microfold cells, which are situated within the epithelium overlying Peyer’s patches. Probiotics can also be directly processed by dendritic cells present in the intestinal lumen and lamina propria. These gut dendritic cells possess the ability to activate naïve CD8? and CD4? T cells, thereby polarizing helper T cell responses toward Th1, Th2, Th17, or Treg profiles. Th1 immune response is predominantly characterized by IFN-γ production and is associated with cell-mediated immunity. Conversely, the Th2 immune response is marked by the secretion of IL-4 and IL-5, which facilitates humoral immunity. The Th17 immune response is defined by the production of IL-17, whereas the induction of Tregs results in the release of IL-10 or TGF-β. Additionally, probiotics can promote maturation of B-cells into IgA-producing plasma cells. Intestinal epithelial cells secrete cytokines and chemokines, establishing a microenvironment within the lamina propria that supports B-cell clonal expansion for IgA production. IgA then gets translocated across the epithelium into the mucus layer, where it regulates bacterial adherence to the host tissues. IgA, immunoglobulin A.

Probiotics are live microorganisms that exert beneficial health effects on the host when consumed in adequate amounts (Liu X. et al., 2025). They have demonstrated potential in modulating intestinal dysbiosis and mitigating the associated ocular complications. The most frequently utilized probiotics predominantly belong to the genera *Lactobacillus* and *Bifidobacterium* (van Lier et al., 2023). These advantageous microorganisms exert their effects through several mechanisms, including modulation of Janus kinase/signal transducer and activator of transcription (JAK/STAT) and NF-κB signaling pathways within intestinal epithelial cells, thereby mitigating inflammatory responses, facilitating tissue repair, and enhancing cellular stress adaptation (Aghamohammad et al., 2022). Additionally, probiotics can regulate various immune cells, including T cells, dendritic cells, and macrophages, to maintain immune homeostasis (Figure 11; Yan and Polk, 2011; Mazziotta et al., 2023).

Probiotics contribute to the enhancement of the intestinal barrier function, which serves as a critical defense against the translocation of harmful substances and bacteria into the systemic circulation. This is achieved by stimulating the secretion of protective intestinal mucus and reinforcing the structural integrity of tight junctions between epithelial cells, thereby markedly improving the gut barrier function (Nagpal et al., 2018). Furthermore, these beneficial microorganisms secrete bacteriocins and other antimicrobial peptides, which are natural bioactive substances that directly inhibit pathogen growth, prevent bacterial translocation, and modulate the expression of pattern recognition receptors. Collectively, these actions promote intestinal homeostasis and immune regulation (Liu et al., 2022).

In experimental murine models, the oral administration of probiotics has been found to possess substantial immunomodulatory properties. Research indicates that probiotics, including *Lactobacillus* and *Bifidobacterium* species, facilitate the differentiation of Tregs within the intestinal mucosa and effectively mitigate inflammatory responses (de Oliveira et al., 2017). Following GM depletion through antibiotic treatment, the introduction of a multi-strain probiotic formulation, IRT-5, comprising *Lactobacillus casei*, *Lactobacillus acidophilus*, *Lactobacillus reuteri*, *Bifidobacterium bifidum*, and *Streptococcus thermophilus*, significantly alleviated ocular clinical symptoms in EAU mice by reducing the proportion of pathogenic CD8^+^ T cells (Kim et al., 2017). Similarly, treatment of EAU model mice with the live probiotic *E. coli* Nissle 1917 inhibited uveitis progression, enhanced the integrity of the intestinal mucosal barrier, and promoted an anti-inflammatory state; however, the heat-inactivated form of this strain did not demonstrate significant therapeutic efficacy (Dusek et al., 2021). Taken together, these animal experimental findings indicate that oral probiotics exert potent therapeutic effects on EAU by modulating intestinal immune homeostasis, enhancing intestinal mucosal barrier integrity, and suppressing pathogenic immune cell responses.

Although human studies are relatively limited, the existing findings suggest potential clinical applications. For instance, a case report indicated that a patient with refractory AAU who received adjunctive probiotic therapy along with corticosteroids exhibited a favorable clinical response, suggesting that probiotics may help reduce the recurrence rate of AAU (Napolitano et al., 2021a). However, these observations represent preliminary clinical evidence with a very small sample size, and most supporting data are derived from animal models rather than large-scale controlled human trials. Therefore, while probiotics show promise as a potential adjunctive strategy for refractory AAU, further well-designed clinical studies with larger cohorts are required to confirm efficacy, optimal dosing, and long-term safety before routine clinical use can be recommended.

### 8.2 Prebiotics

Prebiotics constitute a category of dietary components that resist host digestion and absorption but selectively enhance the growth and metabolic activity of beneficial intestinal bacteria, such as *Lactobacillus* and *Bifidobacterium* (van Lier et al., 2023.). Prebiotics serve as specific substrates for probiotics (Sharif et al., 2021). Common prebiotics include galacto-oligosaccharides, fructans, inulin, gluco-oligosaccharides, and resistant starch, which are primarily found in plant-based foods rich in dietary fiber (Di Lodovico et al., 2020). Prebiotics can promote the proliferation of beneficial bacteria with anti-inflammatory properties and undergo fermentation by GM to produce metabolites, such as SCFAs, including propionate and butyrate, and have the potential to restore immune homeostasis (De Filippis et al., 2016; Holscher, 2017; Zmora et al., 2019). Animal studies have demonstrated that exogenous supplementation with propionate and butyrate can modulate immune responses and reduce the severity of EAU (Chen et al., 2017; de Oliveira et al., 2017; Nakamura et al., 2017).

However, the efficacy of administering prebiotics alone in the treatment of ocular diseases remains limited. Current interventional strategies that combine probiotics with prebiotics, referred to as synbiotics, have demonstrated enhanced therapeutic outcomes in patients with uveitis, effectively improving clinical symptoms and reducing inflammatory markers. In a case report, participants received a composite capsule containing fructo-oligosaccharides and seven probiotic strains administered orally twice daily. After 7 months of treatment, patients exhibited improvements in AU and significant reductions in serum inflammatory markers, including C-reactive protein, high-sensitivity C-reactive protein, and erythrocyte sedimentation rate (Askari and Moravejolahkami, 2019). Notably, this evidence is derived from a case report with an extremely small sample size and lacks a rigorous controlled design. This indicates that synbiotic therapy, which leverages the synergistic effects of probiotics and prebiotics, may help treat AU by controlling pro-inflammatory processes, thereby providing a novel and feasible approach for the clinical management of gut-eye axis-related ocular diseases. Nevertheless, further well-controlled clinical studies with larger cohorts are necessary to confirm efficacy, establish optimal regimens, and evaluate long-term safety before synbiotics can be recommended as a routine clinical strategy for the management of gut-eye axis-related ocular diseases.

### 8.3 Antibiotics

The therapeutic potential and implications of antibiotics for EAU have attracted considerable scholarly attention. As previously discussed, oral antibiotics have been found to mitigate ocular inflammation in EAU mouse models by modulating the gut microbiome composition. Specific agents, such as minocycline (Zhou et al., 2020) and berberine (Du et al., 2020), have demonstrated notable efficacy in ameliorating EAU, primarily through mechanisms involving the suppression of retinal microglial activation and enhancement of the intestinal microenvironment. Nonetheless, using antibiotics as monotherapy has certain limitations. The treatment efficacy may be constrained by the complexity and diversity of gut bacterial communities, and the application of broad-spectrum antibiotics can increase the risk of developing drug-resistant strains. Zárate-Bladés et al. (2017) evaluated the effects of ampicillin, metronidazole, neomycin, and vancomycin on restricting the microbial spectrum. The results indicated that although minor changes occurred during disease progression, none of these antibiotics individually reduced the severity of uveitis as effectively as their combined use (Zárate-Bladés et al., 2017). This finding suggests that the bacterial sources contributing to antigenic cross-reactivity are not confined to a single microorganism but involve multiple microbial communities in uveitis pathogenesis. Additionally, the timing of administration is critical; antibiotic treatment administered 1 week prior to EAU induction produced the most favorable outcomes, whereas concurrent administration or long-term treatment demonstrated limited or no efficacy (Horai et al., 2015; Salvador et al., 2023).

Excessive or inappropriate antibiotic use can promote the development of antimicrobial resistance (Lomakin et al., 2023), posing a significant challenge for sustainable treatment strategies. Therefore, developing targeted narrow-spectrum agents that can precisely eliminate specific bacterial populations is essential. Unlike broad-spectrum antibiotics, immunoglobulin A (IgA) can selectively modulate the gut microbiome. For instance, IgA can bind pathogenic bacteria such as *E. coli*, while sparing beneficial species such as *L. casei* (Okai et al., 2016). In experimental colitis models, oral administration of IgA monoclonal antibodies effectively prevents disease progression, highlighting a promising novel therapeutic approach for IBD (Okai et al., 2017). In conclusion, using antibiotics in uveitis management requires careful consideration to balance efficacy with microbiome preservation. Although antibiotics can help control infections and may indirectly promote the growth of beneficial bacteria, their associated risks, such as the development of antibiotic resistance and disruption of GM, cannot be overlooked. Such perturbations can lead to dysbiosis, potentially exacerbating inflammatory responses and worsening the disease outcomes. Research is needed to further clarify the mechanisms through which antibiotics affect microbiomes and to develop strategies to minimize the adverse consequences of antibiotic-induced dysbiosis.

### 8.4 Immunomodulatory drugs

Immunomodulatory agents used to treat non-infectious uveitis not only directly suppress ocular inflammation but also exert indirect therapeutic effects by modulating the gut microbiome. For instance, sulfasalazine has been found to reduce vascular permeability and improve clinical outcomes in joint diseases and HLA-B27-associated uveitis (Deshpande et al., 2023). In patients with VKH disease, cyclosporine A-based immunosuppressive therapy alleviates dysbiosis and mitigates intraocular inflammation, indicating the microbiota-modulating effect of this drug (Ye et al., 2020). In EAU mouse models, low-dose methotrexate diminished cellular immune responses and induced structural changes in the gut microbiome, whereas mycophenolate mofetil increased Treg proportions, reduced effector T-cell activity, and induced significant alterations in microbial composition (Llorenç et al., 2022). These findings suggest that the therapeutic effects of immunomodulatory drugs are closely associated with specific modifications of the GM.

Moreover, biological agents such as adalimumab and infliximab have demonstrated the capacity to modulate the GM in conditions including Crohn’s disease (Zhou et al., 2018; Ribaldone et al., 2019) and AS (Chen Z. et al., 2021). Responders to these therapies often exhibit a restored microbial balance, characterized by a reduction in pathogenic bacteria, such as *Proteobacteria*, and an enrichment of beneficial taxa, including *Lachnospiraceae*. In the clinical trial identified as NCT03828019, the efficacy of adalimumab was evaluated against conventional immunosuppressants in patients diagnosed with non-infectious intermediate, posterior, or panuveitis who required immunosuppressive therapy. The findings revealed that adalimumab exhibited superior corticosteroid-sparing rates at 6 months and higher corticosteroid discontinuation rates at 12 months. Additionally, it facilitated a more rapid reduction in corticosteroid use. The efficacy of adalimumab in enabling the safe tapering of corticosteroids in patients with uveitis may be linked to the baseline composition of the patients’ gut microbiota (Jabs et al., 2025). These results suggest that the gut microbiome has the potential to serve as a predictive biomarker for therapeutic responses.

### 8.5 Bacteriophage therapy

As an emerging biological approach, bacteriophage therapy offers considerable potential for modulating the gut microbiome in the management of non-infectious uveitis. This strategy enables highly specific targeting and lysis of pathogenic bacteria, while preserving the commensal microbial community, thereby promoting intestinal microecological homeostasis. Studies have demonstrated that phage-based interventions are effective in eliminating drug-resistant infections (Zhang et al., 2021). Additionally, bacteriophages have been proposed as therapeutic candidates for ocular conditions such as ulcerative keratitis (Santos et al., 2011), and their efficacy has been validated in models of bacterial endophthalmitis and keratitis. Notably, lysin PlyB phage has demonstrated promising outcomes in preclinical studies (Mursalin et al., 2023). This further confirms that bacteriophages and their lysins are safe and effective therapeutic candidates for combating severe, antibiotic-resistant ocular infections.

Bacteriophages are distinguished not only by their ability to selectively eradicate pathogenic bacterial populations but also by their potential to modulate the overall composition of the gut microbiome (Kurilovich and Geva-Zatorsky, 2025). Compared to broad-spectrum antibiotics, phages exhibit greater specificity and favorable biosafety profiles in human applications (Gordillo Altamirano and Barr, 2019). Their precise microbial regulatory capabilities provide novel insights into the treatment of microbiota-associated diseases and offer promising avenues for intraocular drug delivery. Although research on phage therapy for uveitis remains limited, the ongoing identification of uveitis-specific microbial markers highlights the potential of developing targeted phage therapies. Future studies aimed at demonstrating the mechanistic interactions among bacteriophages, GM, and uveitis will be essential to advance this therapeutic strategy for clinical application.

### 8.6 FMT

Fecal microbiota transplantation is a medical procedure in which functional microbial communities are isolated, purified, and prepared from the stool of healthy donors and then transplanted into the gastrointestinal tract of patients (Nguyen et al., 2025). This intervention aims to restore intestinal microecological balance and address specific diseases. Currently, FMT is approved for the treatment of recurrent *Clostridium difficile* infections (Hvas et al., 2019). Emerging research suggests that FMT may also have therapeutic potential for conditions such as dry eye syndrome (Watane et al., 2022). Although these studies are limited in scale and often rely on subjective reports of symptom improvement, they provide a rationale for exploring the application of FMT for the management of autoimmune ocular diseases. In a study by Ye et al. (2018), fecal microbiota from patients with BD was transplanted into B10RIII mice, resulting in a marked exacerbation of EAU. RT-PCR analyses confirmed increased production of inflammatory cytokines, including IL-17 and IFN-γ, in the spleen. Similarly, Parker et al. (2022) reported that transplantation of GM from young mice into aged mice improved retinal health in older recipients. Collectively, these studies illustrate that FMT exerts a pivotal regulatory effect on ocular pathophysiology. However, most supporting evidence is derived from animal models and mechanistic studies, revealing a notable scarcity of clinical data on FMT in conditions such as IBD and uveitis. Moreover, gut microbiome dysbiosis patterns in patients with uveitis may differ according to disease subtype, suggesting that FMT requires “precision matching” of donor microbiota, rather than a simple transfer of broadly beneficial bacteria. The clinical translation and widespread implementation of FMT for ocular autoimmune diseases necessitate further research and rigorous investigation.

### 8.7 Dietary intervention

Dietary intervention has emerged as a promising adjuvant strategy for the treatment of uveitis, characterized by low toxicity and feasibility, particularly through its modulation of GM dysbiosis and immune imbalance. Based on existing animal experimental evidence, several key dietary patterns have been identified as effective in mitigating ocular inflammation. Notably, a diet enriched with fermentable fibers, such as pectin, resistant starch, and inulin, is the most substantiated approach. This dietary pattern facilitates the fermentation process of GM, leading to the production of short-chain fatty acids like acetate and propionate, which in turn promote the proliferation of Tregs and inhibit the activity of pathogenic Th1 and Th17 cells. Concurrently, it induces a restructuring of the GM, characterized by an increased abundance of beneficial bacteria, including *Bacteroides*, *Bifidobacterium*, and *Akkermansia*, and a reduction in pro-inflammatory bacteria such as *Desulfovibrio*. Among these dietary interventions, a high-pectin diet exhibits the most pronounced protective effects, significantly lowering clinical uveitis scores, enhancing gut morphology, and ameliorating increased intestinal permeability (Nakamura et al., 2023). The ketogenic diet, characterized by its high-fat and low-carbohydrate composition, has been shown in research by Duan et al. (2024) to rectify the imbalance between Th17 and Treg cells in the retina, reduce the pathogenicity of CD4+ T cells, and mitigate inflammatory infiltration in the retinal tissue. Additionally, moderate caloric restriction, defined as a reduction in total caloric intake by 20%–30%, suppresses glycolysis in pro-inflammatory immune cells, facilitates the expansion of Treg cells, inhibits the proliferation of Th1/Th17 cells, and alleviates EAU, while avoiding the detrimental effects of excessive caloric restriction on immune function (Li et al., 2024). In conclusion, a diet rich in fermentable fibers is recommended as a more suitable long-term adjuvant therapy due to its established safety and efficacy, whereas ketogenic diets and caloric restriction necessitate personalized implementation. Future clinical trials are warranted to validate the synergistic effects of these dietary interventions in conjunction with conventional pharmacological treatments, thereby providing precise guidelines for the management of uveitis.

## 9 Conclusion

Recently, the interplay between the gastrointestinal system and ocular health has attracted increased scholarly attention. Although the gut has traditionally been studied primarily for its role in nutrient metabolism, it is now recognized as a host for a vast and diverse microbial community that engages in a dynamic symbiotic relationship with the immune system. This interaction is essential for maintaining gastrointestinal homeostasis and for modulating systemic inflammatory processes. Consequently, GM imbalance has been linked to a wide range of systemic health issues, including ocular disorders such as uveitis.

Research on the gut-eye axis has revealed significant connections between gut microbial dysbiosis and uveitis. Evidence from experimental models and clinical observations indicates that alterations in microbial composition and function can modulate systemic immune responses, potentially initiating or exacerbating intraocular inflammation. The key mechanisms underlying this relationship include molecular mimicry, compromised intestinal barrier integrity, microbial metabolite signaling, and disruption of gut immune homeostasis, particularly through SCFAs. Collectively, these findings indicate that the gut microbiome is a critical factor in uveitis pathogenesis and highlight its potential as a target for innovative therapeutic strategies.

Although compelling correlations between GM and uveitis have been established, causal relationships remain to be fully validated. Future research should prioritize longitudinal human cohort studies and mechanistic experiments to demonstrate temporal and functional links between specific microbial taxa and uveitis subtypes. Concurrently, interventional strategies, including probiotics, prebiotics, bacteriophage therapy, and FMT, offer promising microbiota-targeted approaches. As these insights progress, translating gut microbiome research into clinical practice may pave the way for novel personalized therapies for this sight-threatening inflammatory disease.
